# The Most-Cited Authors Who Published Papers in JMIR mHealth and uHealth Using the Authorship-Weighted Scheme: Bibliometric Analysis

**DOI:** 10.2196/11567

**Published:** 2020-05-07

**Authors:** Wei-Chih Kan, Willy Chou, Tsair-Wei Chien, Yu-Tsen Yeh, Po-Hsin Chou

**Affiliations:** 1 Department of Nephrology Chi Mei Medical Center, Taiwan Tainan Taiwan; 2 Department of Biological Science and Technology Chung Hwa University of Medical Technology Tainan Taiwan; 3 Department of Physical Medicine and Rehabilitation Chi Mei Medical Center Tainan Taiwan; 4 Department of Physical Medicine and Rehabilitation Chung Shan Medical University Taichun Taiwan; 5 Department of Medical Research Chi Mei Medical Center, Taiwan Tainan Taiwan; 6 Medical School St George’s, University of London London United Kingdom; 7 Department of Orthopedics and Traumatology Taipei Veterans General Hospital Taipei Taiwan; 8 School of Medicine National Yang-Ming University Taipei Taiwan

**Keywords:** betweenness centrality, authorship collaboration, Google Maps, social network analysis, knowledge concept map, the author-weighted scheme

## Abstract

**Background:**

Many previous papers have investigated most-cited articles or most productive authors in academics, but few have studied most-cited authors. Two challenges are faced in doing so, one of which is that some different authors will have the same name in the bibliometric data, and the second is that coauthors’ contributions are different in the article byline. No study has dealt with the matter of duplicate names in bibliometric data. Although betweenness centrality (BC) is one of the most popular degrees of density in social network analysis (SNA), few have applied the BC algorithm to interpret a network’s characteristics. A quantitative scheme must be used for calculating weighted author credits and then applying the metrics in comparison.

**Objective:**

This study aimed to apply the BC algorithm to examine possible identical names in a network and report the most-cited authors for a journal related to international mobile health (mHealth) research.

**Methods:**

We obtained 676 abstracts from Medline based on the keywords “JMIR mHealth and uHealth” (Journal) on June 30, 2018. The author names, countries/areas, and author-defined keywords were recorded. The BCs were then calculated for the following: (1) the most-cited authors displayed on Google Maps; (2) the geographical distribution of countries/areas for the first author; and (3) the keywords dispersed by BC and related to article topics in comparison on citation indices. Pajek software was used to yield the BC for each entity (or node). Bibliometric indices, including h-, g-, and x-indexes, the mean of core articles on g(Ag)=sum (citations on g-core/publications on g-core), and author impact factor (AIF), were applied.

**Results:**

We found that the most-cited author was Sherif M Badawy (from the United States), who had published six articles on JMIR mHealth and uHealth with high bibliometric indices (h=3; AIF=8.47; x=4.68; Ag=5.26). We also found that the two countries with the highest BC were the United States and the United Kingdom and that the two keyword clusters of mHealth and telemedicine earned the highest indices in comparison to other counterparts. All visual representations were successfully displayed on Google Maps.

**Conclusions:**

The most cited authors were selected using the authorship-weighted scheme (AWS), and the keywords of mHealth and telemedicine were more highly cited than other counterparts. The results on Google Maps are novel and unique as knowledge concept maps for understanding the feature of a journal. The research approaches used in this study (ie, BC and AWS) can be applied to other bibliometric analyses in the future.

## Introduction

### Background

As of April 12, 2018, more than 146 papers were found by the keyword “author collaboration” (Title), 1168 by “author collaboration,” and 53 by “author collaboration” and “bibliometric” in the Medline Library. A phenomenal increase has been found in the number of research papers with multiple authors [1]. The knowledge of discovery is no longer contained merely in the departments of a local university but in an international article author byline [2]. Increasing academic pressure and prestige-concerned individuals with prolific publications have also been forced to claim authorship for many aspirants on paper publications [3]. Given academic developments in recent years, the features of author collaboration on one topic or for a specific journal should be investigated.

### Issue of Duplicate Authors in a Network

An author’s publication features can be determined by social network analysis (SNA) [4-8]. However, no study currently in the literature describes the issue of duplicate names in bibliometric data, which might result in biases because some different authors with the same name exist [7]. For instance, authors [7] stressed that:

[T]here might be some biases of understanding for author collaboration because some different authors with the same name or abbreviation exist, who are affiliated to different institutions. The result of author relationship analysis for mHealth research would be influenced by the accuracy of the indexing author.

Three main centrality measures (ie, degree, closeness, and betweenness) are frequently used to evaluate the influence (or power) momentum of an entity (or the author of a study) in a network [9,10]. Few studies have applied betweenness centrality (BC) to interpreting a network’s characteristics. In this study, we aimed to explore whether BC can solve the problem of detecting duplicate authors in a network.

### Issue of Most-Cited Authors in a Given Journal

As of June 31, 2020, over 269 articles were found by searching the keyword “most cited” (Title) in PubMed Central (PMC) and 39 papers by “most productive author” or “most prolific author.” However, few had studied most-cited authors. The reason might be that there is no quantitative scheme that has been successfully used to calculate weighted author credits in the literature; even many counting schemes have been proposed for quantifying coauthor contributions [11-13]. Thus, an authorship-weighted scheme (AWS) will be required for application to bibliometric metrics to allow for comparison.

### Issue of a Dashboard Possibly Shown on Google Maps

The author’s publication patterns are always presented with static .jpg format pictures [4-7] instead of a dynamic dashboard that allows readers to see further details on their own. We have observed many bibliometric studies [7,14-19] using coword (or coauthor) analysis to visualize study data. However, no work has displayed their findings with a zoom-in and zoom-out functionality on Google Maps [20,21]. A breakthrough in showing data on Google Maps is a worthwhile task to develop.

### Objectives

The journal of JMIR mHealth and uHealth was targeted for BC algorithm application to examine possible duplicate authors with the same names in a network. Our goal is to select the most highly cited authors in author collaborations. Also, both features (ie, the affiliation regions distributed for the first author in geography, and the keywords related to article topics) will be investigated using the citation analysis in this study.

## Methods

### Data Collection

When searching the PubMed database (Pubmed.org) maintained by the US National Library of Medicine, we used the keywords “JMIR mHealth and uHealth” (Journal) on June 30, 2018. We then downloaded 676 articles that had been published since 2013, because the first article in JMIR mHealth and uHealth was published in 2013. An author-made Microsoft Excel (Microsoft Corporation, Albuquerque, New Mexico, United States) VBA (visual basic for applications) module was used to analyze the research data. All downloaded abstracts were based on the type of journal article involved. Ethical approval was not necessary for this study because all the data were obtained online from the Medline library.

### Social Network Analysis and the Betweenness Centrality

SNA [22] was applied to explore the pattern of entities in a system using the software Pajek [in Koeln; PajekMan in Osoje (Ossiach, Austria)] [23]. In keeping with the Pajek guidelines, we defined an author (or paper keyword) as a node (or an actor) that is connected to other nodes through the edge (or the relation). The number of connections usually defines the weight between two nodes.

Centrality is a vital index for analyzing a network. Any individual or keyword in the center of a social network will determine its influence on the network and its speed at gaining information [9,24]. In this study, we used the BC, which may be defined loosely as the number of times a node needs a given node to reach another node [9,25], as in, the number of shortest paths passing through a given node. The BC is expressed as follows, in Standalone Equation 1:

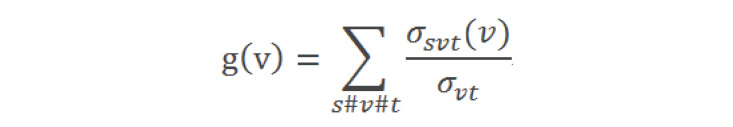



By contrast, the BC of node v, which is denoted as g(v), is obtained as svt in Standalone Equation 1. The BC of node v is the number of shortest paths from node s to node t (s,t≠v). Finally, the BC should be divided by the possible number of connected nodes, (N-1)(N-2)/2, where N is the number of nodes in the network. If all the nodes go through v in the shortest path, g(v) is equal to 1.

**Figure 1:**
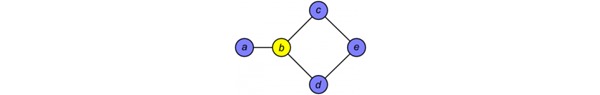
Calculation of betweenness centrality.

The BC for node b is calculated in Figure 1 and Standalone Equation 2.

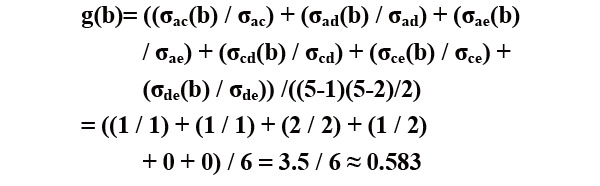



The two nodes (ie, a and e) have two equal shortest paths (ie, abce and abde). The number of shortest paths from node a to node e is 2.

The method used to ensure there are no authors with duplicate names in the network is to identify the large bubble (with high BC) by clicking the linked coauthors and checking if the author is identical between any two neighbor subnetworks (see Multimedia Appendix 1 and 2).

### The Author-Weighted Scheme

The AWS and the author impact factor (AIF) calculations are shown in Standalone Equations 3 and 4:

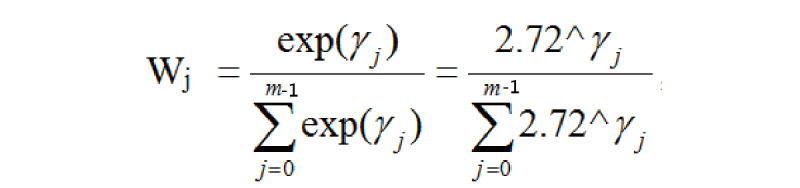

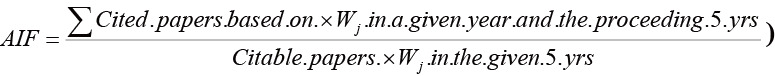





Considering a paper of m+1 authors with the last being the corresponding author, W_j_ denotes the weight for an author on the order j in the article byline. The power, γ_j_, is an integer number from m–1 to 0 in descending order. The sum of author weights in a byline is Standalone Equation 5.

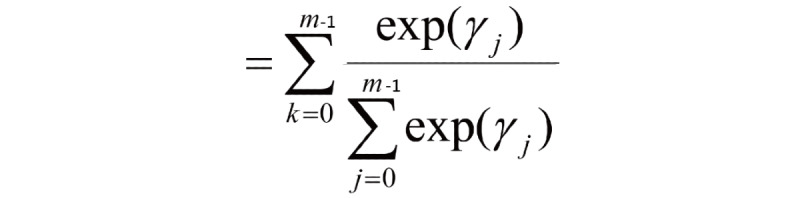



The sum of authorships equals 1 for each paper referred to in Standalone Equation 5. This is a basic concept ensuring that all papers have an equal weight irrespective of the number of coauthors [26]. Accordingly, more importance is given to the first (exp[m], primary) and the last (exp[m–1], corresponding or supervisory) authors, whereas it is assumed that the others (the middle authors) have made smaller contributions [27,28]. In Standalone Equation 5, the smallest portion (exp(0)=1) is assigned to the last second author with the odds=1 as the basic reference [29,30].

### Pattern of Author and Nation Collaboration in JMIR mHealth and uHealth

We selected JMIR mHealth and uHealth as the target journal. The authors (n1=3522) (see Multimedia Appendix 3) were collected. The most cited authors using citation analysis were plotted on Google Maps. Bibliometric indices, including the h-, g-, and x-indexes [31-33], the mean of core articles on g(Ag) (citations on g-core/publications on g-core), and the AIF [34,35] for representing individual research achievements were used to evaluate authors and article topics (ie, the keyword clusters). The most highly cited authors can be plotted with a dashboard on Google Maps using the Kano diagram [36,37] to display it. The authors’ x-indexes are located on the X-axis, the h-index is on the Y-axis, and the bubbles are sized by AIF and colored by type within four dragrants (ie, from I to IV denoted by the fearure of excellence, citation-oriended, low performance, and production-oriended, respectively). It is worth noting that the Kano diagram separates all authors into three parts (ie, the h-index originated excitement, the one-dimension performance, and the x-index-originated achievement) [36,37].

The countries/areas of authors for each published paper were extracted to show the distribution of countries/areas on Google Maps using choropleth maps [38]. The darker regions indicate the most pivotal (or influential) role or bridge in the network if the BC algorithm is performed. Furthermore, the top ten keyword clusters were particularly extracted by SNA, and the representatives with the highest BC in their respective clusters were highlighted on Google Maps. SNA thus filtered the author-defined keywords (n2=1678). Details about the graphical process using SNA and Google Maps are illustrated in Multimedia Appendices 4 and 5.

## Results

### The Most Cited Authors Shown on Google Maps

**Figure 2:**
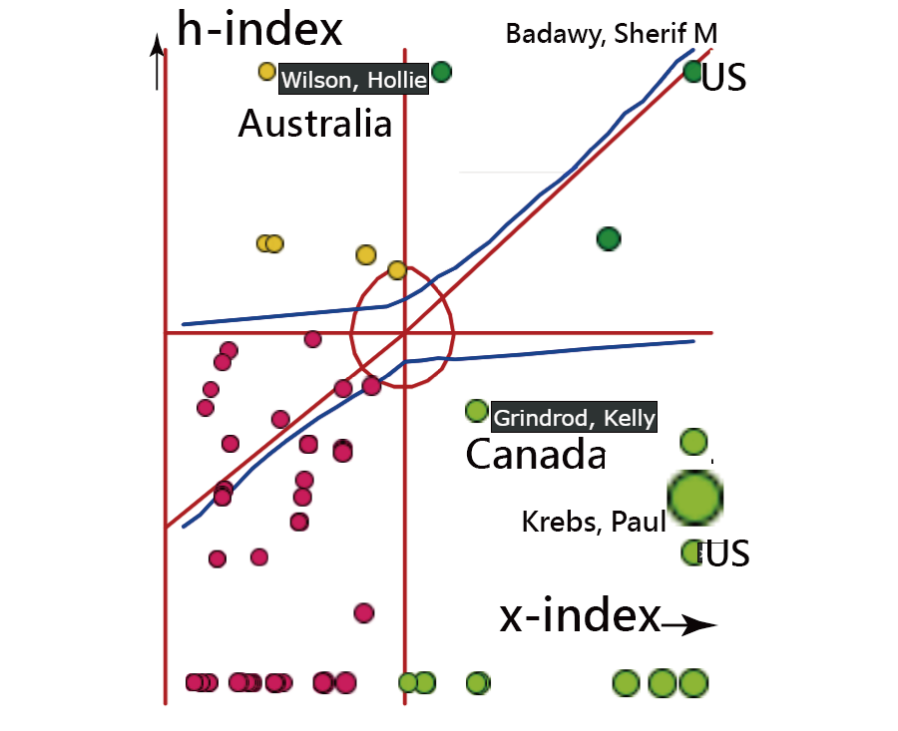
Authors’ citations dispersed on Google Maps.

The most-cited author is Sherif M Badawy (from the United States), who published six articles on JMIR mHealth and uHealth with high bibliometric indices (h=3; AIF=8.47; x=4.68; Ag=5.26). His top five weighted citations are 9.5 ,7.6, 7.3, 1.3, and 0.5, which yield an h-index of 3 at the third position due to the fourth cited value (1.3) being less than the paper number of 4. The Ag (5.26) and x-index (4.68) are yielded because of g being at 5 (ie, the total citations (26.29) are greater than 25) and x at 3 [ci = 7.3 when computing 
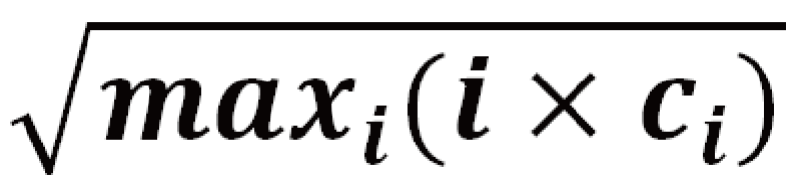
], respectively. The biggest bubble denotes the author Paul Krebs from the United States, who has the highest AIF because one of his articles [39] was cited 178 time in the past. Interested authors can scan the QR-code in Figure 2 [40] to examine the various authors’ publication outputs and details in PMC by clicking the bubble of a specific author.

### Pattern of Countries/Areas Distributed by the First Author

**Figure 3:**
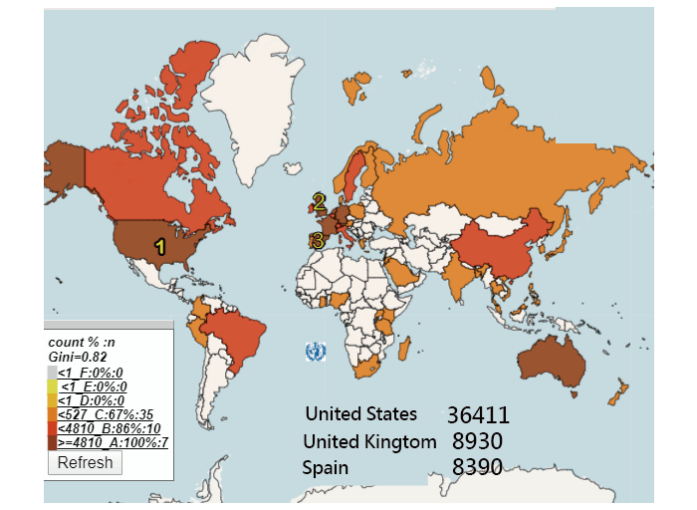
Dispersion of country/area on author collaborations for JMIR mHealth and uHealth.

Figure 3 [41] shows the county/area distribution on Google Maps, indicating most “bridge” coauthors are from two countries, the United States and the United Kingdom, using the BC algorithm.

The top six countries with the highest increase in number of production outputs (ie, Growth>0.90) were the United States, the United Kingdom, South Korea, Canada, Australia, and New Zealand (Table 1). The top two countries with the highest proportion of papers produced were the United States (36.83%) and Australia (9.47%). The x-indexes for each country/area are present in the last column in Table 1. It is worth noting that the x-index for JMIR mHealth and uHealth is 26.56, as shown in the bottom right corner.

**Table 1:** Dispersions of author collaboration across continents over the years

Continent, Country		2013	2014	2015	2016	2017	2018	Total, n (%)	Growth^a^	x-index
**Africa**		—^b^	2	1	2	2	1	8 (1.18)	0.71	—
	Kenya	—	—	1	—	—	—	1 (0.15)	—	1.95
	Nigeria	—	—	—	—	1	—	1 (0.15)	0.71	—
	South Africa	—	2	—	2	1	—	5 (0.74)	0.32	2.42
	Uganda	—	—	—	—	—	1	1 (0.15)	—	—
**Asia**		3	10	8	9	22	32	84 (12.43)	0.83	—
	China	2	2	1	1	7	12	25 (3.7)	0.57	3.19
	South Korea	—	—	2	2	4	6	14 (2.07)	0.94	3.08
	Singapore	—	3	—	—	1	4	8 (1.18)	–0.12	3.56
	Thailand	—	2	2	—	1	2	7 (1.04)	—	2.25
	Taiwan	—	—	—	1	2	3	6 (0.89)	0.88	1.39
	Others	1	3	3	5	7	5	24 (3.55)	0.97	—
**Europe**		15	12	18	35	60	67	207 (30.62)	0.89	—
	United Kingdom	2	—	9	9	13	12	45 (6.66)	0.91	6.65
	Germany	2	2	1	2	11	11	29 (4.29)	0.68	5.97
	Spain	5	1	1	4	5	10	26 (3.85)	0.23	5.41
	Netherlands	1	—	1	9	7	6	24 (3.55)	0.81	4.7
	Sweden	—	3	4	4	3	4	18 (2.66)	0.67	4.84
	Others	5	6	2	7	21	24	65 (9.62)	0.71	—
**North America**		6	21	52	70	90	54	293 (43.34)	0.99	—
	United States	6	17	42	58	79	47	249 (36.83)	0.99	17.13
	Canada	—	4	10	12	11	7	44 (6.51)	0.92	8.74
**Oceania**		1	9	15	21	19	11	76 (11.24)	0.93	—
	Australia	1	8	13	17	15	10	64 (9.47)	0.91	11.03
	New Zealand	—	1	2	4	4	1	12 (1.78)	0.97	4.81
**South America**		—	3	1	—	3	1	8 (1.18)	0.31	—
	Brazil	—	2	—	—	2	1	5 (0.74)	0.29	2.52
	Colombia	—	1	—	—	—	—	1 (0.15)	–0.35	1.59
	Peru	—	—	1	—	1	—	2 (0.3)	0.58	1.59
Total		25	57	95	137	196	166	676 (100)	0.99	26.56

^a^Growth based on data from 2013 and 2017.

^b^Not applicable.

### Clusters of Keywords

**Figure 4:**
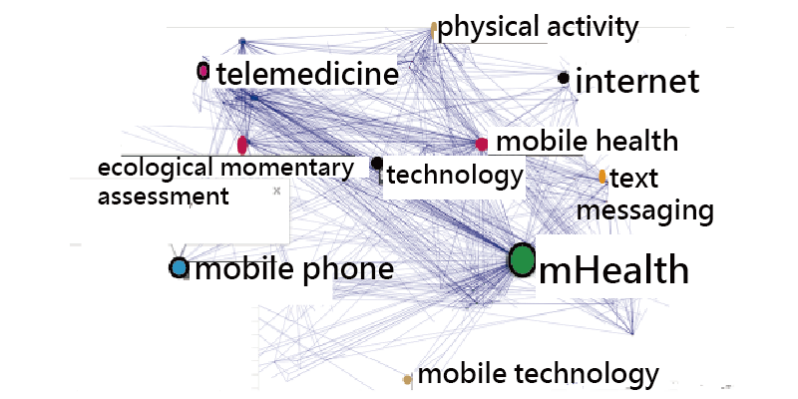
Dispersion of keyword clusters for the first author clusters of JMIR mHealth and uHealth. mHealth: mobile health.

The top ten keyword clusters are presented in Figure 4. The representative terms with the highest betweenness centrality are shown for each cluster. The biggest one is that of “mHealth.” It is recommended that interested readers should scan the QR-code in Figure 4 [42] to see the details of the information on Google Maps.

### Analyses of Article Topics Related to Bibliometric Indices

**Figure 5:**
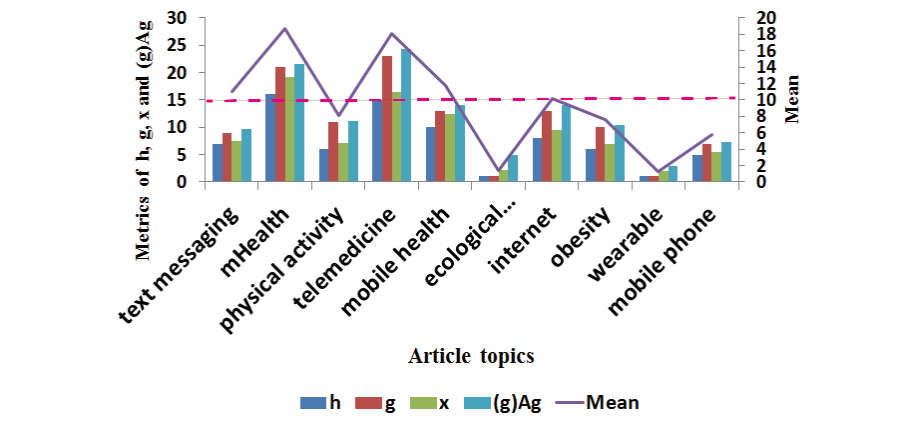
Comparison of article topics related to bibliometric indices. Ag: publication on g-core.

The numbers of citable and cited articles across the keyword clusters are shown in Tables 2 and 3. Five bibliometric indices are present at the right-hand side. We found that the AIF had a weak relation with the other four indices, as shown in the bottom right side in Table 2. However, the journal impact factor is 4.37, equivalent to the impact factor of journal citation report (JCR IF)=4.541 in 2017. The two keyword clusters of mHealth and telemedicine earned the highest indices in comparison to their counterparts (Figure 5), indicating both topics have a higher metric (ie, the normalized mean of h, g, x, and Ag) than the other topic clusters.

**Table 2:** Bibliometric indices for medical subject heading (MeSH) terms over the years for publications.

Keywords	Publication count							AIF^a^	h	g	x	(g)Ag^b^
	2013 (n)	2014 (n)	2015 (n)	2016 (n)	2017 (n)	2018 (n)	Total (N)					
Text messaging	—^c^	4	4	5	6	6	25	4	7	9	7.48	9.67
mHealth^d^	7	16	39	51	68	55	236	4.4	16	21	19.13	21.57
Physical activity	2	3	4	8	16	14	47	2.83	6	11	7.21	11.18
Telemedicine	2	11	18	33	57	51	172	4.87	15	23	16.43	24.26
Mobile health	3	8	9	14	21	15	70	4.6	10	13	12.41	14.08
Ecological momentary assessment	—	—	1	2	2	1	6	1.17	1	1	2.24	5
Internet	3	4	6	3	5	4	25	7.36	8	13	9.54	14
Obesity	1	2	5	8	4	1	21	5.9	6	10	6.93	10.4
Wearable	—	—	1	—	1	3	5	1	1	1	2	3
Mobile phone	1	2	2	6	3	2	16	3.56	5	7	5.48	7.29
Others	6	7	6	6	13	10	48	2.63	—	—	—	—
Total	25	57	95	136	196	162	671	4.37	—	—	—	—

^a^AIF: author impact factor.

^b^(g)Ag: publications on g-core.

^c^Not applicable.

^d^mHealth: mobile health.

**Table 3:** Correlation coefficients of metrics for medical subject heading (MeSH) terms over the years for quantity of citations.

Keywords	Publication count							Correlation	AIF^a^	h	g	x	(g)Ag^b^
	2013 (n)	2014 (n)	2015 (n)	2016 (n)	2017 (n)	2018 (n)	Total (N)						
Text messaging	—^c^	28	28	30	14	0	100	AIF	1	—	—	—	—
mHealth^d^	112	212	335	242	131	7	1039	h	0.57	1	—	—	—
Physical activity	25	18	19	48	23	0	133	g	0.63	0.98	1	—	—
Telemedicine	46	182	307	186	95	22	838	x	0.54	0.99	0.96	1	—
Mobile health	11	82	91	100	38	0	322	Ag	0.58	0.98	0.99	0.96	1
Ecological momentary assessment	—	—	2	5	0	0	7	—	—	—	—	—	—
Internet	33	57	81	9	4	0	184	—	—	—	—	—	—
Obesity	16	12	59	25	12	0	124	—	—	—	—	—	—
Wearable	—	—	3	—	2	0	5	—	—	—	—	—	—
Mobile phone	7	10	25	15	0	0	57	—	—	—	—	—	—
Others	20	35	46	23	2	0	126	—	—	—	—	—	—
Total	270	636	996	683	321	29	2935	—	—	—	—	—	—

^a^AIF: author impact factor.

^b^(g)Ag: publications on g-core.

^c^Not applicable.

^d^mHealth: mobile health.

## Discussion

### Principal Findings

We found that the most-cited author is Sherif M Badawy (from the United States), who has published six articles on JMIR mHealth since 2016. Other authors also gained excellent citation indices on Figure 2, such as Stoyan R Stoyanov from the United States (4 papers since 2015), John Torous from Germany (5 papers since 2014), Paul Krebs from Germany (3 papers since 2014), and Kathryn Mercer from Germany (3 papers since 2015). It is easy to examine their publications on PubMed by clicking the author’s bubble on Google Maps.

The most productive authors with six papers were Urs-Vito Albrecht (citable=2.6; cited=18.1; AIF=6.8) from Germany, and Sherif M. Badawy (citable=3.3; cited=27.7; AIF=8.5) from the United States. The reason why Badawy has a higher weighted value of citable papers than Albrecht is that the latter was the middle author more often than the former if the AWS in Standalone Equation 3 was applied. If the BCs were applied, the author Ralph Maddison, from Australia, who had five papers (citable=1.1; cited=6.1; AIF=5.5), played the most pivotal (bridge) role in the authoring network.

The two countries with the highest BC were the United States (x-index=17.13) and the United Kingdom (x-index=6.65), thereby proving that the United States and Europe still dominate publication output in science [43,44]. Another new finding is about the two keyword clusters of mHealth and telemedicine with the highest metrics among types of article feature, which is rarely seen when combining citation analysis and SNA in previous articles.

### Strength of the Study

Traditionally, in dealing with a test with multiple questions and answers, we often count the item with the highest frequency as representing the most important value. For instance, many customers purchase their goods in a shopping cart, which is like a test of multiple answers without considering any associations between entities. Accordingly, many articles [4-8] merely present the highly frequency counts of authors instead of the association of authors in a network, such as the most productive authors Urs-Vito Albrecht and Sherif M. Badawy in Figure 2, instead of the most pivotal author Ralph Maddison with the highest BC, who is associated with many coauthors in the network. Many data scientists have developed ways to discover new knowledge from the vast quantities of increasingly available information [45], especially by applying SNA [4-6] to large data analysis.

**Figure 6:**
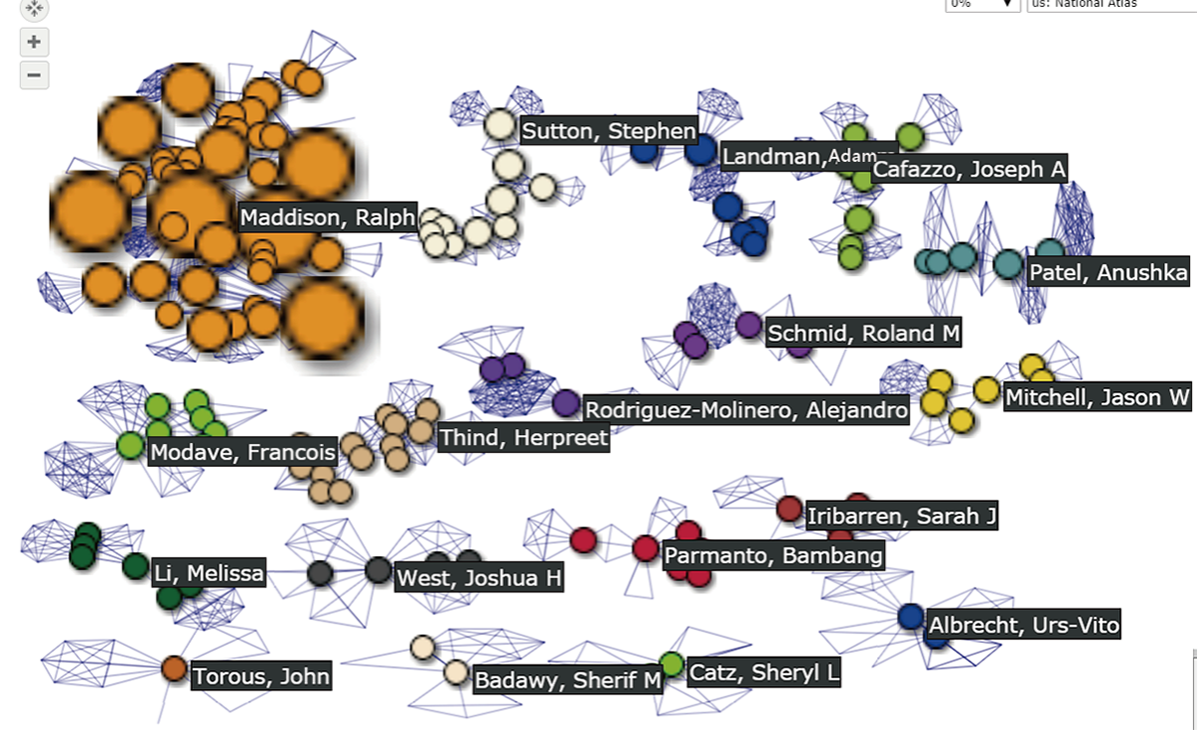
Author clusters in a collaboration network.

We also ensured that no author had duplicate names in the network via identification of the large bubble (ie, with a high BC) first by clicking the linked coauthors (eg, Francois Modave at the left-bottom bubble in Figure 6), and then checking the author without duplicate names in the network by clicking the associated coauthors in the opposite neighbor subnetworks to examine whether the author had the same names in each paper. The dashboard [46] could easily be linked to the published papers in Medline if the author was clicked. For further details about the steps made to ensure there were no authors without duplicate names, see Multimedia Appendices 1 and 2.

Furthermore, we found 335 papers in Medline because of the keyword social network analysis (Title) as of May 20, 2018. In practice, we found studies on duplicative prescriptions using SNA in Japan [47] and one explaining HIV risk multiplexity [48]. However, no such study like ours has incorporated the SNA analysis with Google Maps to interpret the results. Many papers investigated most-cited articles or most productive authors in academics. Few inspected most-cited authors in a given journal. Overall, two challenges we faced have been overcome in this study: (1) some different authors with the same name in bibliometric data; and (2) coauthors’ contributions differing in the article byline. Furthermore, we illustrated a way to examine article topics associated with the number of citations for a journal.

Previous studies [49-51] reported: (1) a higher impact factor being associated with the publication of reviews and original articles instead of case reports; (2) rigorous systematic reviews receiving more citations than other narrative reviews; and (3) case reports with low impact factors due to them being rarely cited by articles. In comparison, we applied the author-defined keywords to cluster article features, which is different from previous studies in that an objective verification was made for a given journal. As such, the bibliometric metrics can be linked to the article features if each article has been assigned to its corresponding type.

Regarding the incorporation of Google Maps with SNA, Google Maps are sophisticatedly linked in references [41-52] for readers interested in manipulating the link as a dashboard. The country/area distribution in Figure 3 easily illustrates the feature of international author collaborations in JMIR mHealth and uHealth. We hope subsequent studies can report other types of information using the Google application programming interface to readers in the future.

### Limitations and Future Study

Although findings were based on the above analysis, the results should be interpreted with caution because of several potential limitations. First, this study only focused on a single journal. Any generalization should be made in similar fields of journal contents. Second, although SNA is quite useful in exploring the topic evolution and identifying hotspots for keywords, the results might be affected by the accuracy of the author-defined terms. The medical subject heading (MeSH) terms included in the PubMed library are recommended for use in the future. Third, many different algorithms are used for SNA. We merely applied community cluster and density with BC in the figures. Any changes made along with the algorithm will present different patterns and inferences. Fourth, SNA is not subject to the Pajek software we used in this study. Others, such as Ucinet [53] and Gephi [54], are suggested to readers for use in the future. Fifth, we downloaded citing articles from PMC, which are different from many citation analyses that use other academic databases, such as the Scientific Citation Index, Scopus, and Google Scholar [55-58], to investigate the most cited articles in a specific discipline. This approach using data from PMC can lead to more citation studies reporting the most cited authors in other disciplines.

### Conclusions

The most cited authors were selected using the authorship-weighted scheme (AWS). The keywords of mHealth and telemedicine are potentially highly cited more than other types of keywords. The results on Google Maps are novel and unique as a knowledge concept maps for understanding the features of a journal. The research approaches used in this study (ie, BC and AWS) can be applied to other bibliometric analyses in the future.

## Appendix

Multimedia Appendix 1 MP4: Identifying the unique author name.

Multimedia Appendix 2 PDF:using between centrality to detect authors with duplicate names in a network.

Multimedia Appendix 3 Txt:Pajek control file and dataset.

Multimedia Appendix 4 MP4”How to deal with data and build the Google maps.

Multimedia Appendix 5 MP4: MS Excel module extracting data from a website and plotting Google Maps.

## Abbreviations

AIF: author impact factor
AWS: authorship-weighted scheme
BC: betweenness centrality
g(Ag): citations on g-core/publications on g-core
MeSH: medical subject heading
PMC: PubMed Central
SNA: social network analysis
VBA: visual basic for applications
