# Supplementary material for: The Most-Cited Authors Who Published Papers in JMIR mHealth and uHealth Using the Authorship-Weighted Scheme: Bibliometric Analysis
Source: JMIR Mhealth Uhealth. 2020 May 7;8(5):e11567. doi: 10.2196/11567 (PMC7319608; doi:10.2196/11567)

Selecting the author who is not duplicated in a network

Background:

We often encounter the situation that author information about the institute or city or nation data incomplete in the study dataset such as in MEDLINE library. How to deal with cleaning data or say identical duplicate authors in the library is required to define.

Rule:

If excluding the author, all others authors related to the one we concern in a network will be connected together with each other in one component.

Decision tree:

Data→(1)Select the Maximal Betweenness Centrality→Remove it and check any two or more separated cluster in existence?

If exists, check whether the two or more separated clusters are identical in relation to the pivotal author?

If related to each other, no any duplicate author exists.

(2) repeat (1) to remove any other maximal Between Centrality which might be service agents (or providers) who are the bridge to connect any two different clusters related together.

(3) if we have the name of the author to check if duplicate name exist, we can use the approach in (1) and (2) to confirm it.

For example:

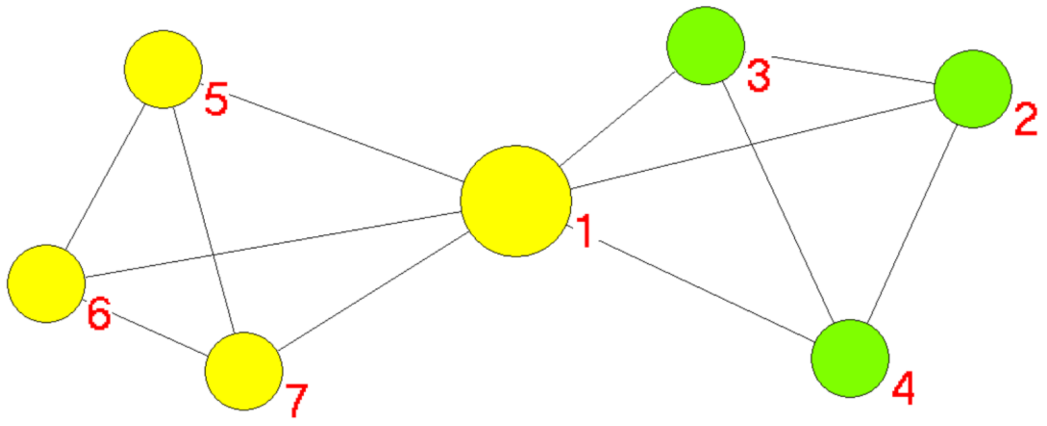

In above Figure, we can see the author 1 plays the pivotal bridge role in the network using the Partitioned Community algorithm to separate and define the clusters.

If the Betweenness Centrality is applied to define the power in cluster, we can see the author 1 has been significantly outlined in the network, see the Figure below.

If we remove the author 1 from the network, it can be seen that the two clusters

have been independently separated from each other. At this time, we check whether the two clusters have identical author attributes, If yes, no any duplicate authors exist. Otherwise, the author 1 has strong evidence that there are two duplicate authors with an identical name.

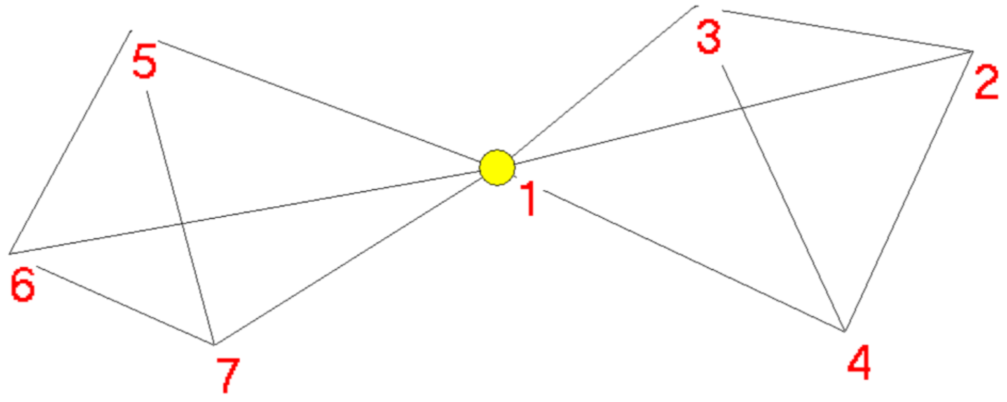

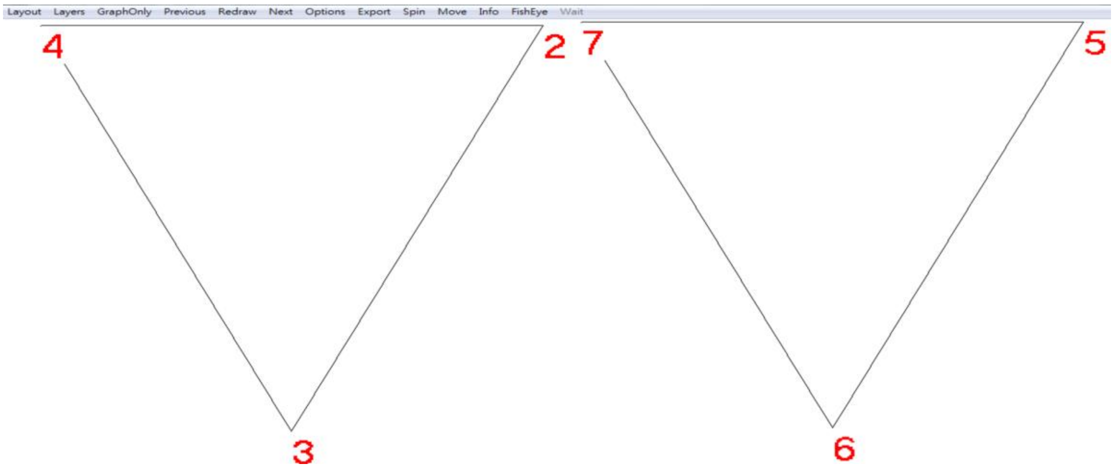

Another scenario is the network that has three with the highest Between Centrality. One might be the duplicate author, other two might be the service agent(or provider such as statistician) for article publications see the Figure below.

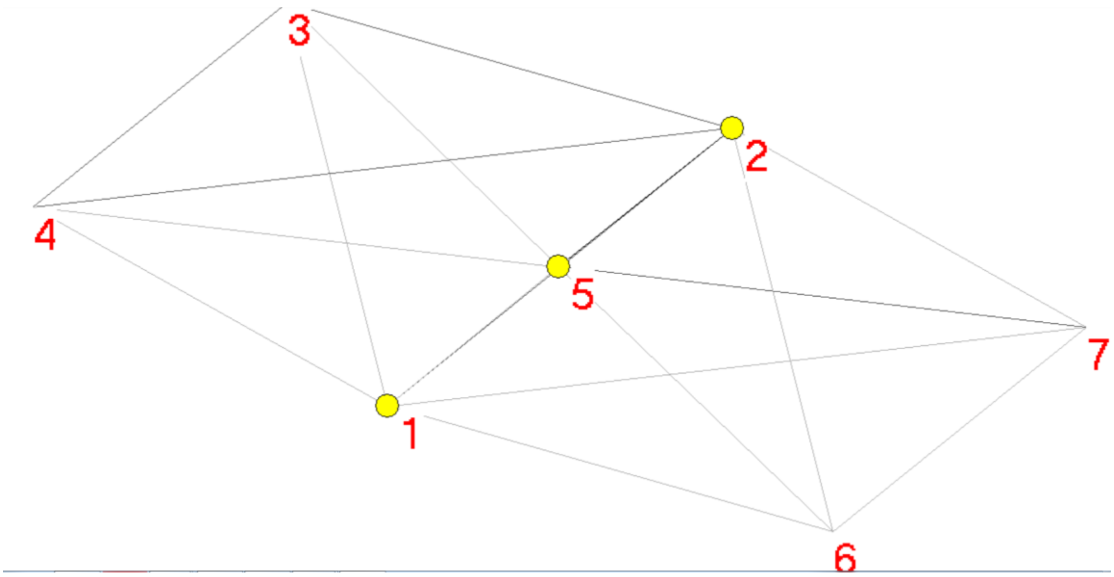

We thus check the network one-by-one on the author with the highest Between Centrality in the network and remove them. We can see the two authors who might be the duplicate author or the service agent. It depends on the fact that any two or more clusters are with an identical author we concern in this network.

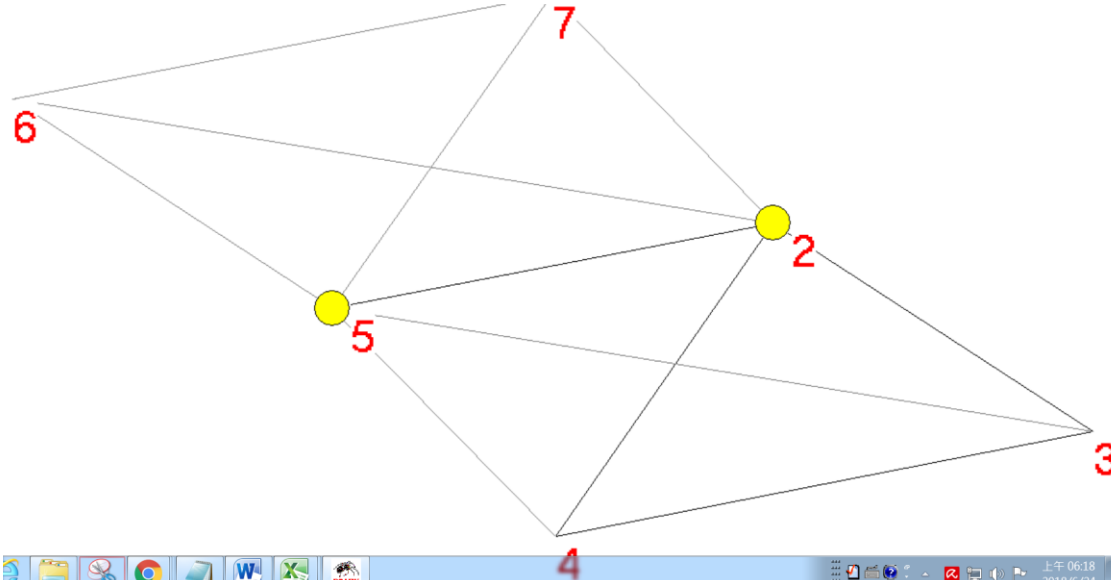

One-by-one to check any special feature in this network by using the highest Betweenness Centrality on the author we concern.

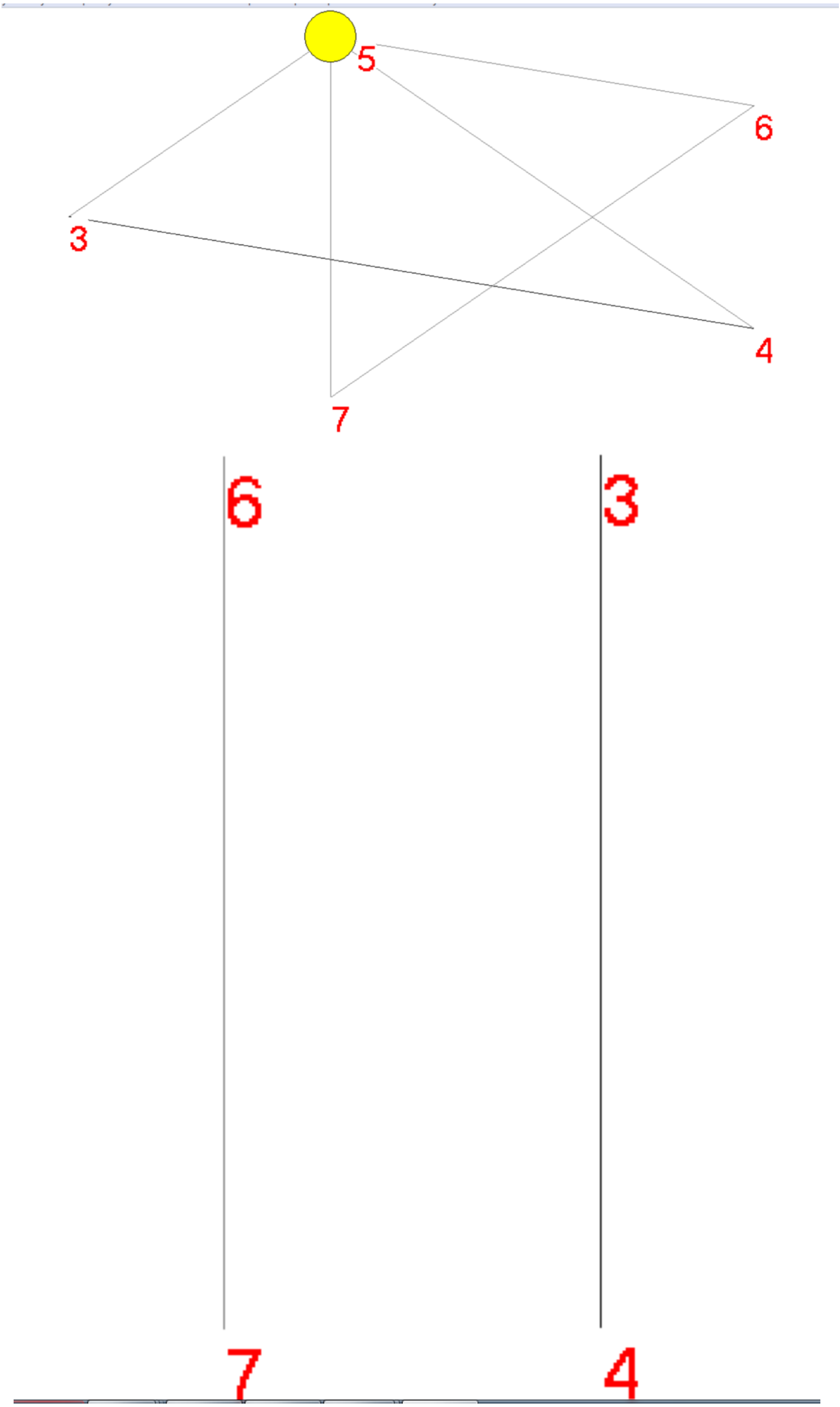
5
6
3
4
7
6
3
7
4

Experiment:

See below all authors but the one named Wang, Wei are independently in a component.

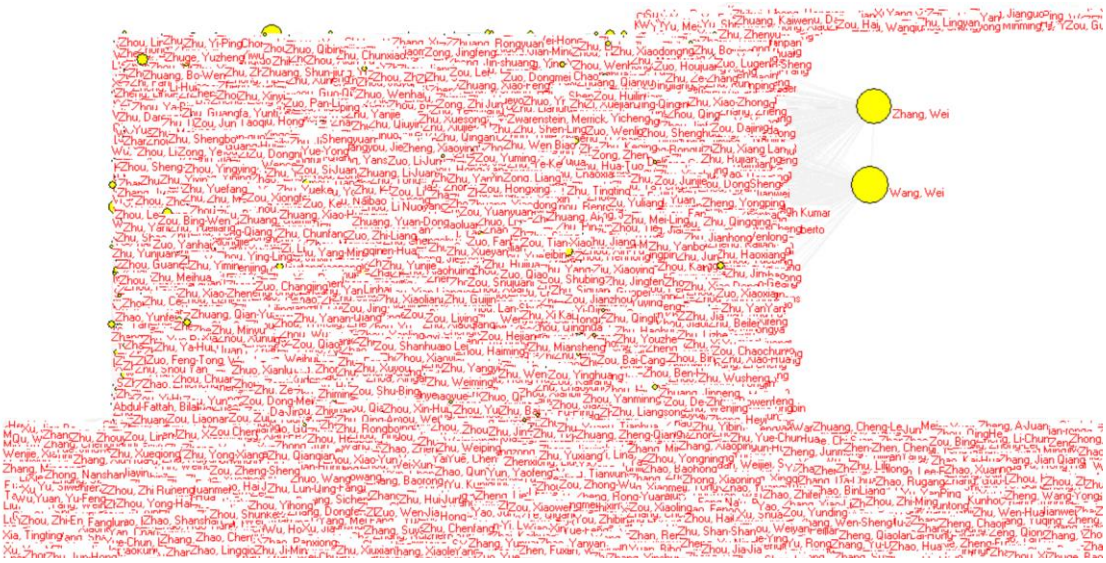

Kao, Chia-Hung

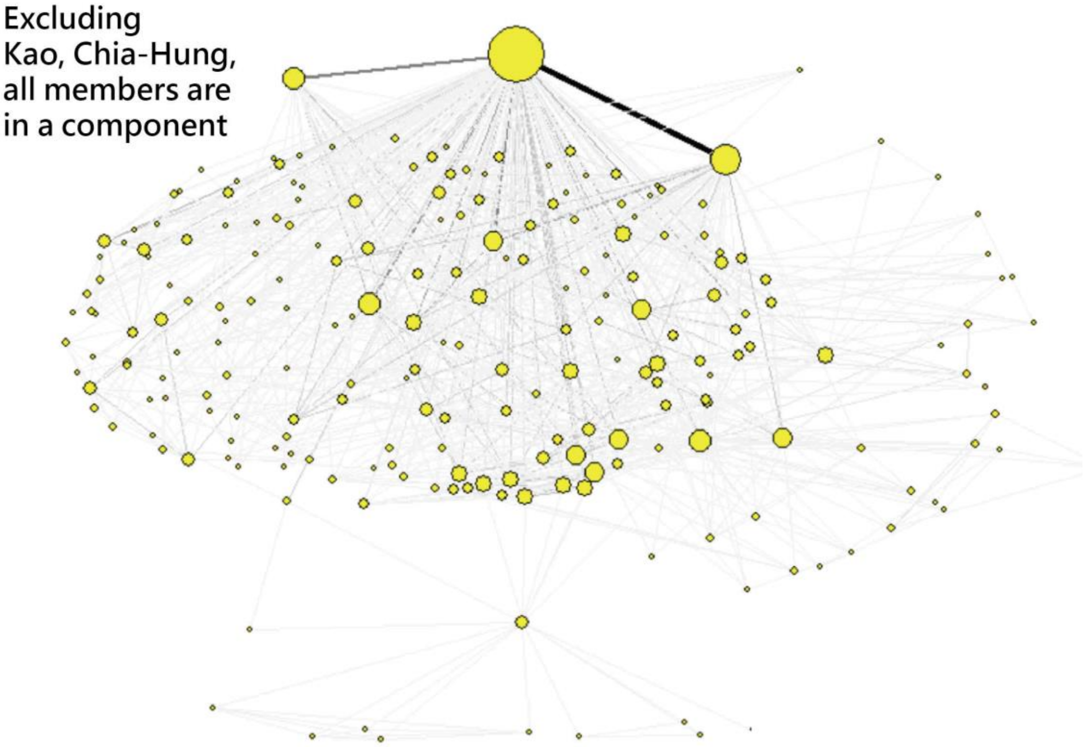

Excluding
Gonzalez-Gay, Miguel A,
all members are
in a component

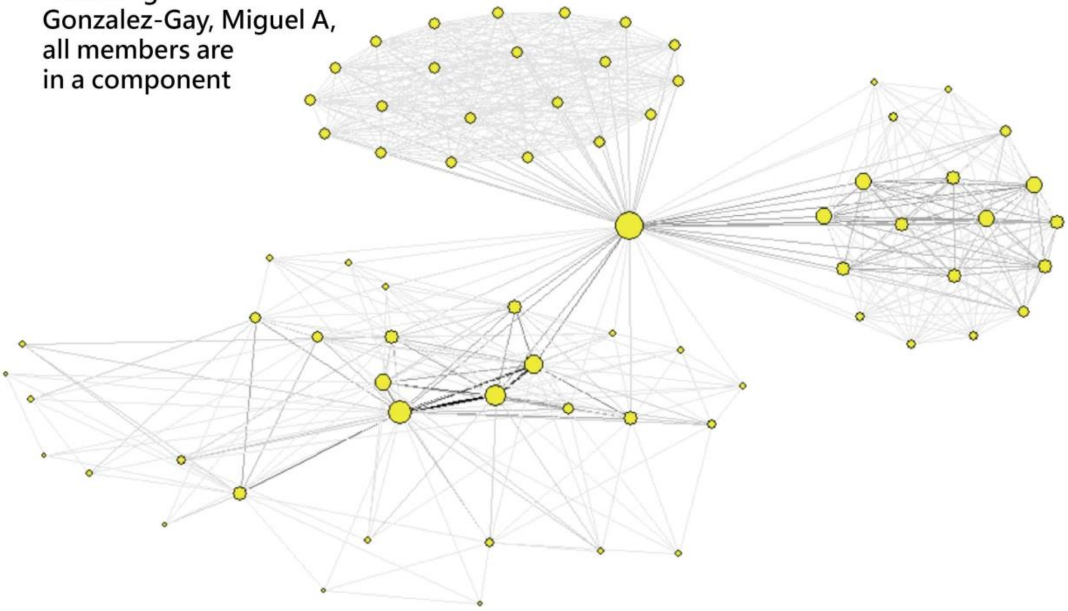

Excluding
Bouza, Emilio,
all members are
in a component

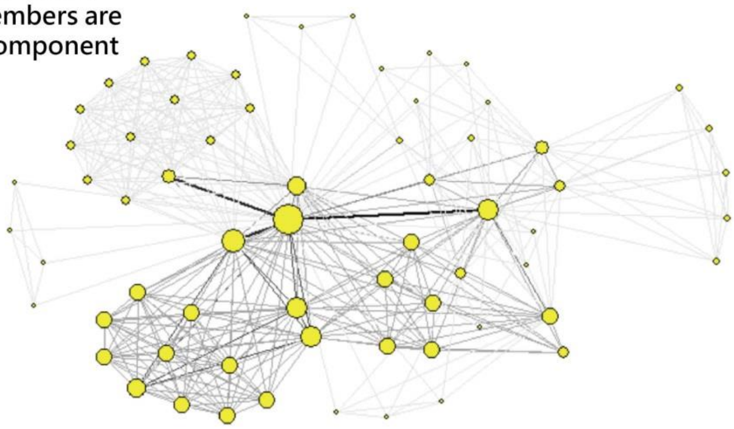

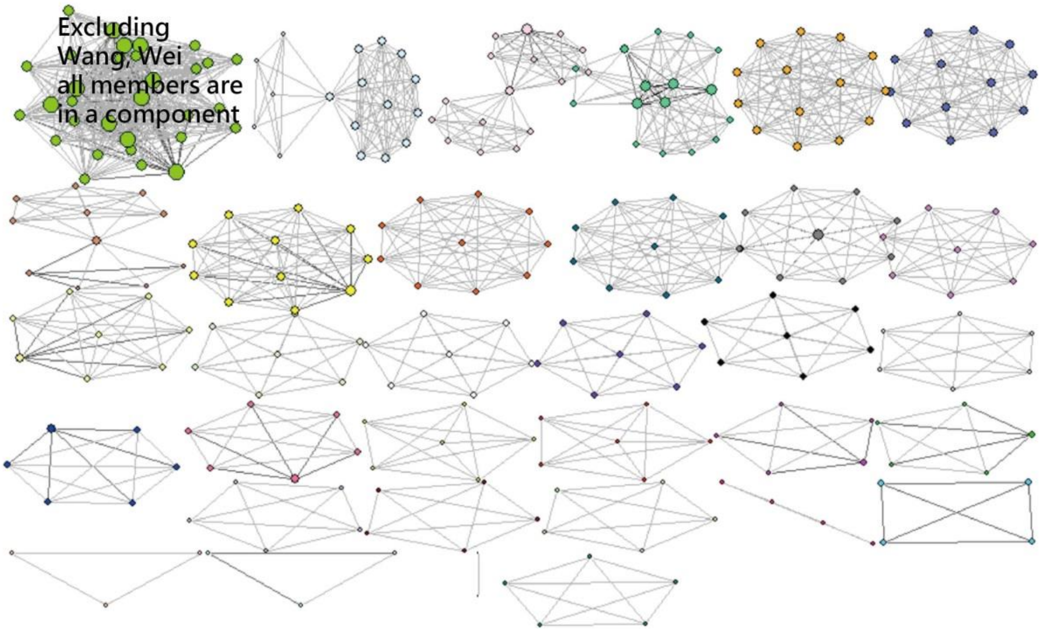
Excluding
Wang, Wei
all members are
in a component

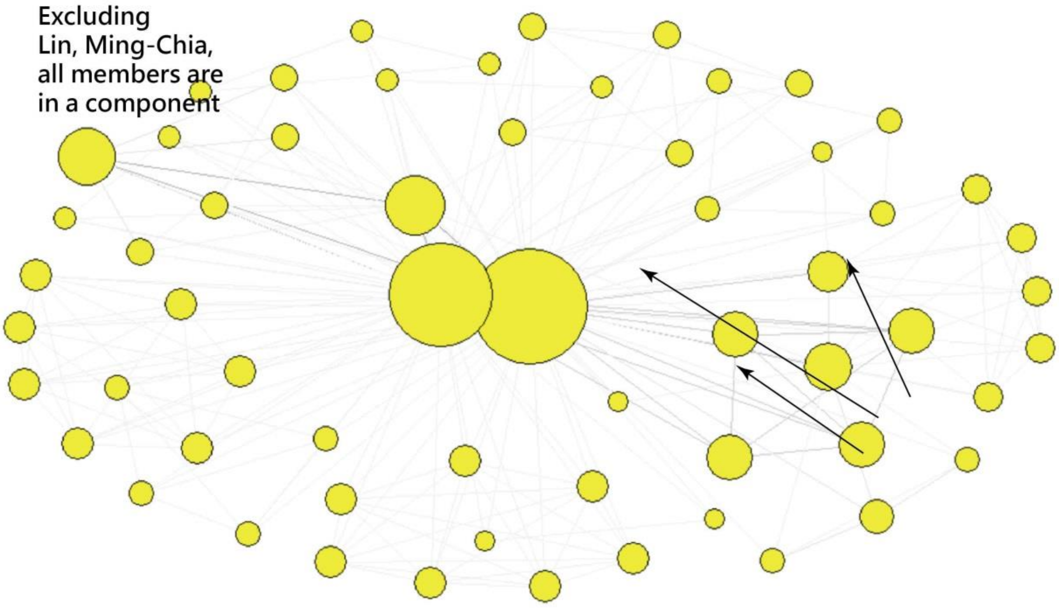
Excluding
Lin, Ming-Chia,
all members are
in a component

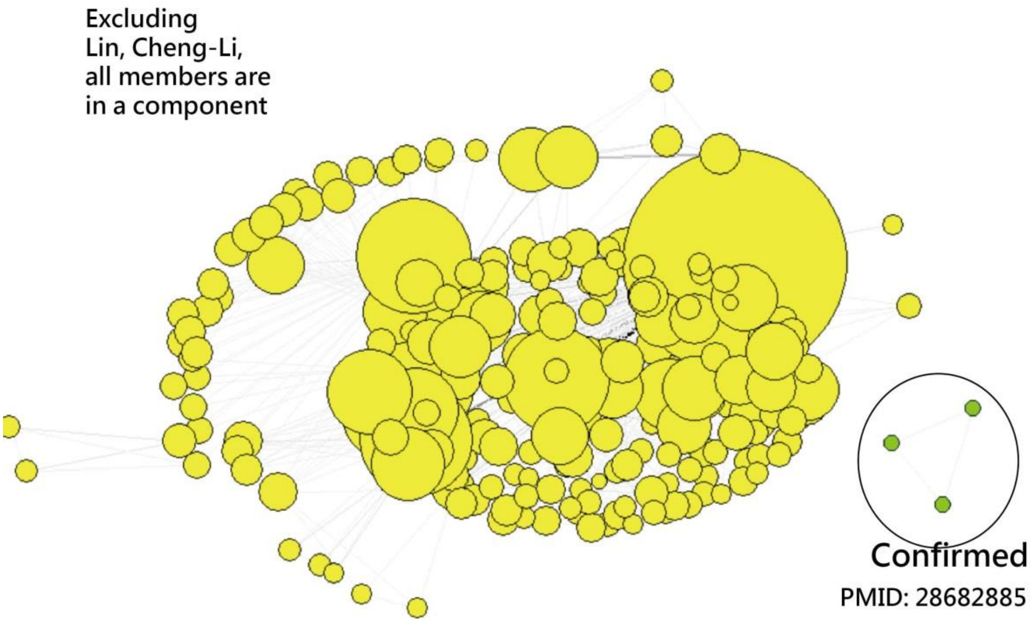
Excluding
Lin, Cheng-Li,
all members are
in a component
Confirmed
PMID: 28682885

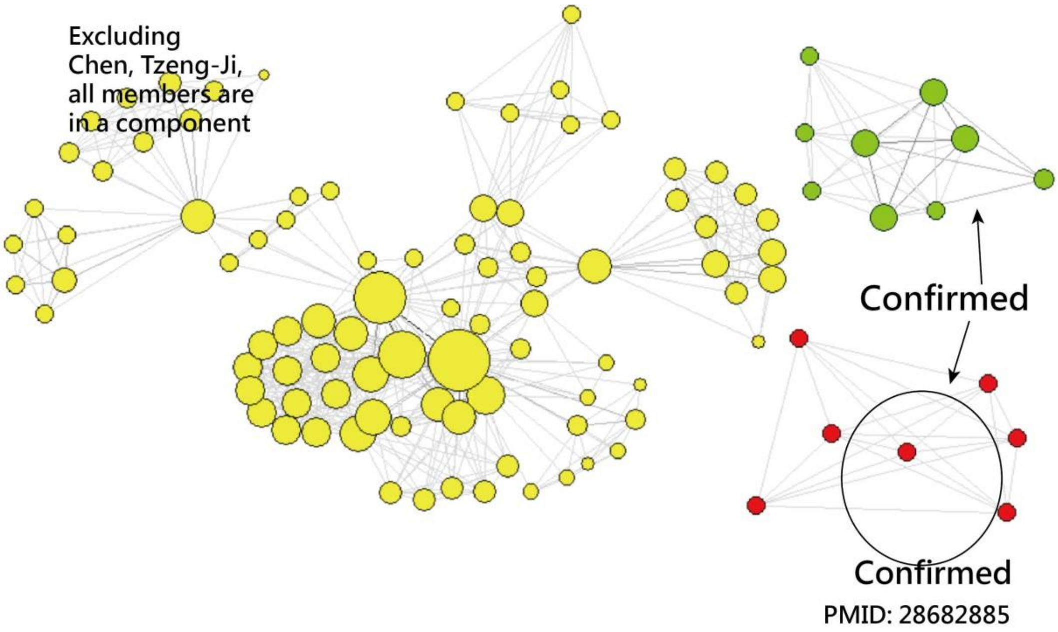
Excluding
Chen, Tzeng-Ji,
all members are
in a component
Confirmed
Confirmed
PMID: 28682885

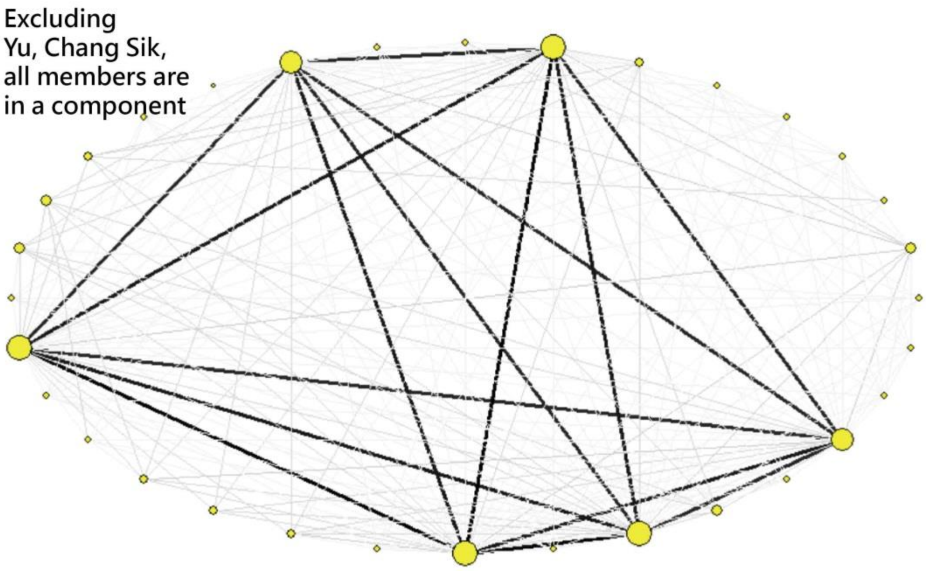
Excluding
Yu, Chang Sik,
all members are
in a component

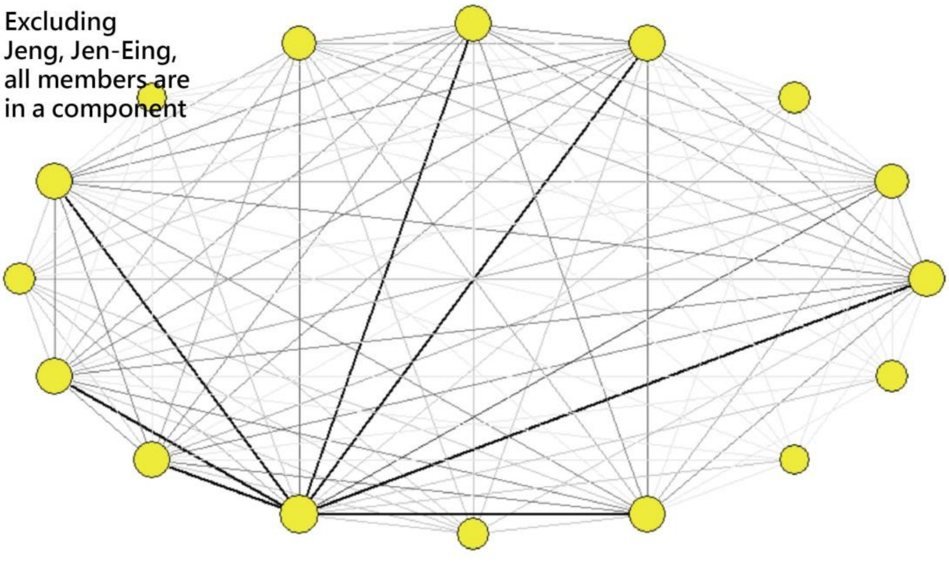
Excluding
Jeng, Jen-Eing,
all members are
in a component

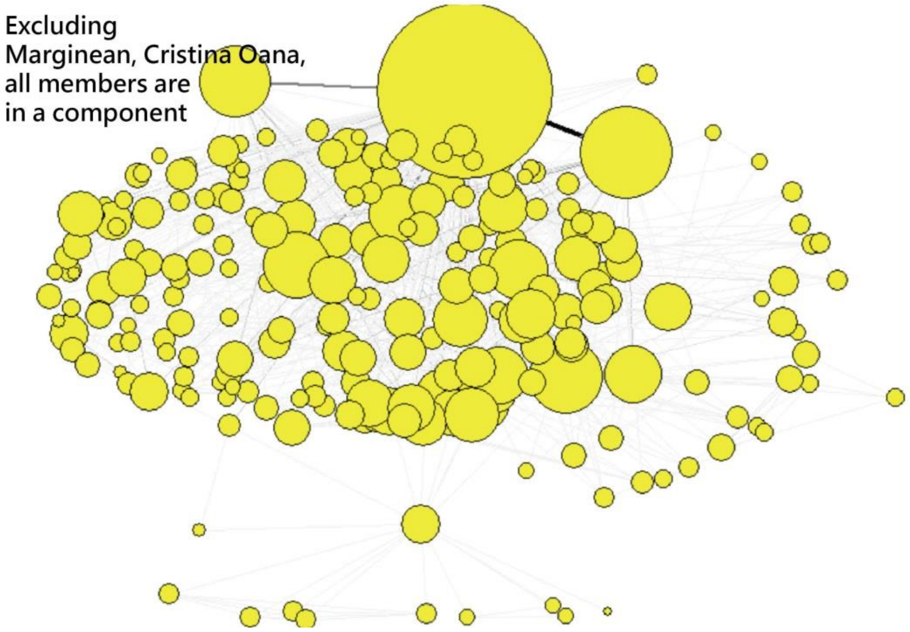
Excluding
Marginean, Cristina Oana,
all members are
in a component

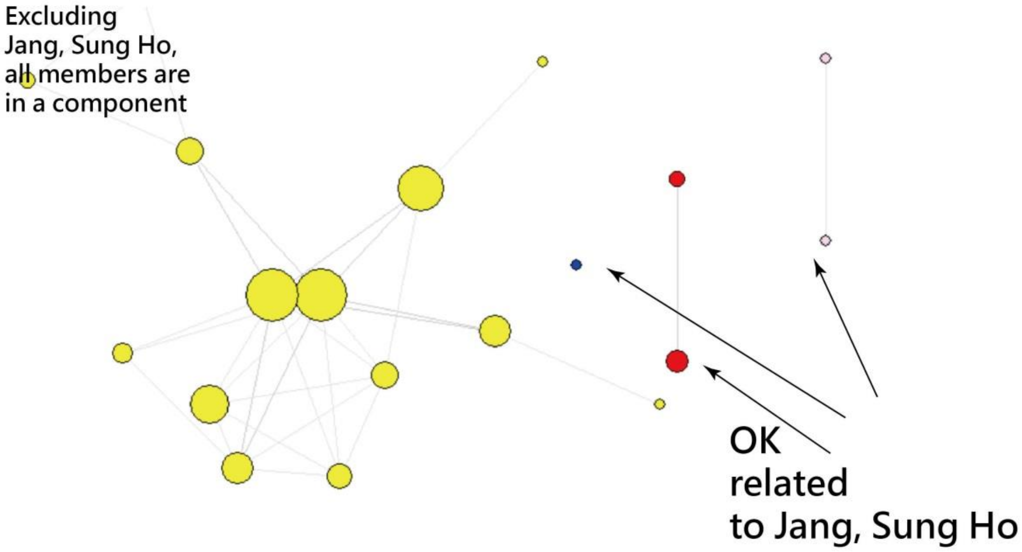
Excluding
Jang, Sung Ho,
all members are
in a component
OK
related
to Jang, Sung Ho

Excluding
Lee, Jacky W Y,
all members are
in a component

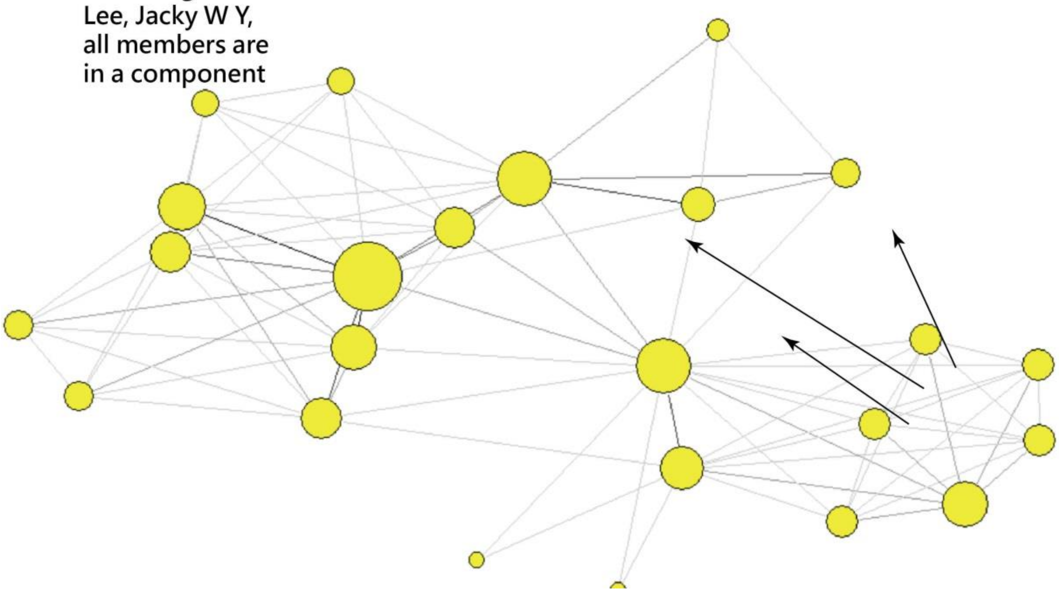

Supplement: Multimedia Appendix 2 [file mhealth_v8i5e11567_app2.pdf]
